# Brucellosis outbreak in a remote village in northwestern Tajikistan in 2023: a matched case-control study

**DOI:** 10.3389/fepid.2024.1470917

**Published:** 2024-10-11

**Authors:** Emomali Qurbonov, Jamila Silemonshoeva, Roberta Horth, Zulfiya Tilloeva, Salomudin Yusufi, Dilyara Nabirova

**Affiliations:** ^1^ Central Asia Advanced Field Epidemiology Training Program, Almaty, Kazakhstan; ^2^ Preventive Medicine, Asfendiyarov Kazakh National Medical University, Almaty, Kazakhstan; ^3^ Department of Epidemiology, HIV Prevention and Control Center of Sighd Region, Khujand, Tajikistan; ^4^ Department of Epidemiology, State Center for Sanitary and Epidemiological Surveillance of Sughd Region, Khujand, Tajikistan; ^5^ U.S. Centers for Disease Control and Prevention, Central Asia Office, Almaty, Kazakhstan; ^6^ Department of Epidemiology, SI “City Disinfection Station”, Dushanbe, Tajikistan; ^7^ Department of Medical and Pharmaceutical Education, Ministry of Health and Social Protection of the Population, Dushanbe, Tajikistan

**Keywords:** disease outbreaks, brucellosis, animals, domestic, case-control studies, milk, meat, Tajikistan

## Abstract

**Background:**

A sharp increase in reported brucellosis incidence was observed in northwestern Tajikistan (from 1.0/100,000 people in January–May 2022 to 32.7/100,000 in January–May 2023). Most (82%) cases were from the same remote mountainous village (population = 10,712). The aim of this study was to identify risk factors for brucellosis infection and mitigate disease risk.

**Methods:**

Using a case-control design, we conducted face-to-face interviews and collected blood samples during May-June 2023. Fifty-seven cases and 114 controls were recruited. Cases were the first person in a household diagnosed with brucellosis during February–June 2023 with positive serum agglutination test and antibody titers ≥1/160 from blood samples. Two controls were selected for each case (neighbors from different households matched by age and sex). Controls testing positive were excluded and replaced. We conducted conditional multivariable logistic regression to calculate adjusted odds ratio (AOR) and 95% confidence intervals (CI).

**Results:**

Among the 87 brucellosis patients reported, 57 (66%) agreed to participate and didn't have secondary cases in the household. Of the 57 cases, 68% were 15–44 years old, and 44% were male. Cases peaked in May 2023. Common symptoms were joint pain (95%), fever (84%), weakness (72%), and night sweats (65%). Of selected controls, 13% tested positive and were excluded. All cases and 94% of controls owned livestock (mostly cattle, sheep, or goats); no animals had not been vaccinated in the past 5 years. Brucellosis was associated with consumption of both homemade kaymak (clotted cream) and home-raised meat compared with neither (AOR: 59 [95%CI: 4.3–798], *p* < 0.01), home-raised meat but not kaymak compared with neither (AOR: 54 [4.0–731], *p* < 0.01), and involvement in animal slaughter compared with no involvement (AOR: 36 [2.8–461], *p* < 0.01).

**Conclusion:**

Contact with unvaccinated livestock or consumption of their products was a key contributor to this outbreak in a remote village of Tajikistan. With 13% of controls testing positive, true incidence was likely greater than reported. Following our investigation, a brucellosis awareness education campaign and animal vaccination campaigns were carried out in the region and only one case was reported in September 2023.

## Introduction

Brucellosis is a common bacterial zoonotic infection characterized by a wide range of non-specific influenza-like clinical manifestations in humans. *Brucella* spp. is transmitted from infected animals such as cattle, goats, and sheep to humans through consumption of raw dairy and meat products or by direct contact with tissues and fluids (1). Human-to-human transmission is rare (2). There are an estimated 500,000 new cases in humans annually (3). The highest burden of disease is in the Middle East and Central Asia. Large outbreaks are regularly documented in these regions, the majority of which are associated with consumption of raw dairy products from unvaccinated animals (4, 5). Although there are no approved vaccines to prevent human brucellosis, vaccination of animals is highly effective in reducing disease (6, 7). Pasteurization of milk destroys *Brucella* spp (8). but is not commonly done in Tajikistan.

Tajikistan ranks among the top 25 countries with the highest incidence of brucellosis (9). During 2005–2014, the average annual incidence of brucellosis in Tajikistan was 14.0 per 100,000 people (10). An estimated 74% of the population in Tajikistan lives in rural areas, with approximately 60% working in agriculture and/or animal husbandry. Brucellosis is considered endemic in most districts in Tajikistan, and the risk is particularly high in regions where the population predominantly works in agriculture and animal husbandry (10–12).

**Figure 1:**
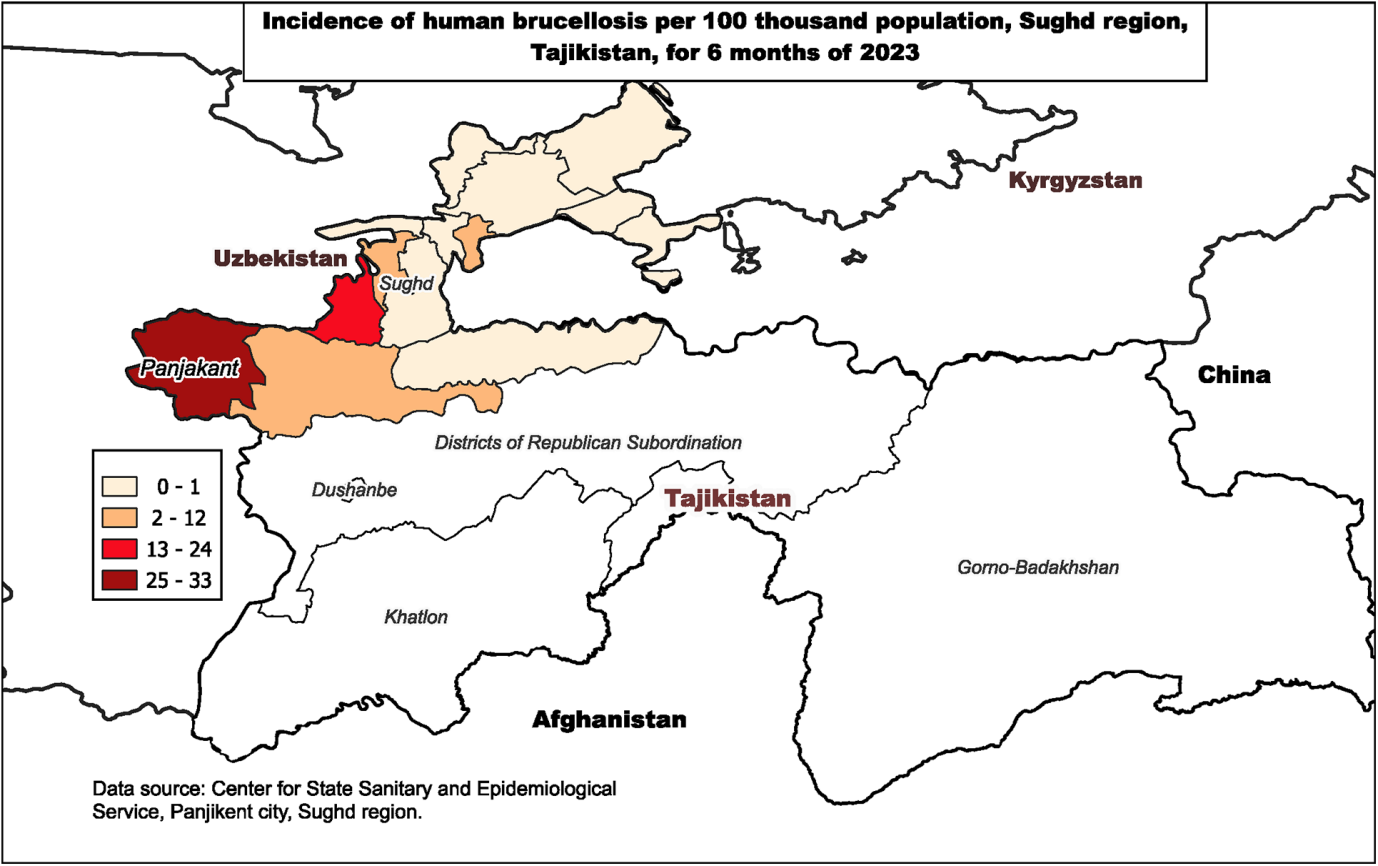
Incidence of human brucellosis per 100,000 population, Sughd region, Tajikistan, January–June 2023.

In May 2023, a sharp increase of brucellosis was observed in a district in northwestern Tajikistan (Figure 1). Incidence in the district was much higher than in surrounding districts. In addition, 12-month incidence in the district in 2022 was 1.0/100,000 people and 5-month incidence by May 2023 had already reached 32.7/100,000. The majority of cases, 84% (87/103), were concentrated in the same remote mountainous village with a population of 10,712.

A rapid response team composed of residents of the Field Epidemiology Training Program, epidemiologists and laboratorians from the Center for State Sanitary and Epidemiological Surveillance from the region, and specialists from the Committee for Food Security conducted an investigation. The aim was to identify risk factors for brucellosis infection and provide recommendations for preventing outbreaks.

## Methods

### Study design

We conducted a case-control study in the affected village in May and June 2023. Cases were defined as the first person in a household diagnosed with brucellosis from January 1 to June 15, 2023. Brucellosis diagnosis was based on: having at least two symptoms (fatigue, fever, joint pain, night sweats, headache), a positive Huddleson reaction test, and Wright antibody titers ≥1/160 from blood samples taken from both cases and controls. Both Huddleson and Wright are serological agglutination tests (13). Cases were diagnosed at healthcare facilities.

Sample size was estimated based on a hypothesis that half of cases would have a direct animal contact exposure compared to one-quarter of controls. Using Fleiss statistical methods for rates and proportions, a sample size of two controls for every included case (57 cases) would be sufficient to detect an odds ratio of 3.0 with 95% confidence interval and 80% power.

Controls were conveniently selected healthy neighbors from a neighboring household matched to a case by age ±5 years. Controls testing positive and those with a history of brucellosis were excluded. For each excluded control, a new control was selected so that each case had two controls for analysis. Cases and controls had all lived more than 6 months in the village.

### Data source

We conducted computer-assisted face-to-face interviews at participant households using a semi-structured questionnaire. The questionnaire included questions related to participants’ socio-demographic characteristics, medical histories, and environmental and behavioral risk factors associated with brucellosis in the literature. This included knowledge of brucellosis transmission, dairy and meat consumption practices, and contact with animals.

For cases, we abstracted data from medical and laboratory records. For controls, we collected blood samples for brucellosis detection using the Huddleson reaction method. Qualitative determination of antibodies to somatic antigens of *Brucella* spp. was performed using a set of reagents from “ECOlab” company (term of use till 07.2024), registered in the Federal Service for Supervision in the Sphere of Health Care of the Russian Federation No. FSR 2008/02480 dated 02.07.2018.

### Ethical considerations

The study was reviewed and approved by the Ministry of Health and Social Protection of Tajikistan (No. 1-5/5271 dated 11.06.2024). Additionally, this activity was reviewed by CDC, deemed not research, and was conducted consistent with applicable federal law and CDC policy.^1^ The study was presented to community members and the study team received verbal endorsement by local leaders who facilitated access to households. Participation was voluntary and anonymous and no personally identifiable information was collected on survey forms. Verbal informed consent was obtained for all participants. Children were not interviewed. For anyone under the age of consent (<18 years old) interviews were conducted with the child's primary caregiver.

### Data analysis

Data were entered using the Kobo Toolbox (Cambridge, USA). Data were cleaned and analyzed using R v.4.3 (The R Foundation, Vienna, Austria). We conducted a bivariable analysis of exposures associated with brucellosis using Chi-square tests. Conditional multivariable logistic regression model was used to assess the association between disease and risk factors. We report adjusted odds ratios and 95% confidence intervals from the final model. Interaction and additive effects between variables were assessed. Wald test was used to determine statistical significance. A *p*-value <0.05 was defined as statistically significant.

## Results

### Study participants

Among 87 brucellosis patients reported from January to June 15 in the village, 57 (66%) were included in this study. Most (*n* = 21) excluded cases were excluded because they were from the same household as another included case; nine cases were excluded due to refusal. Of 131 controls selected for the study, 13% tested positive for brucellosis antibodies, indicating a history of disease, and were therefore excluded; 114 controls were included.

### Characteristics of cases

**Figure 2:**
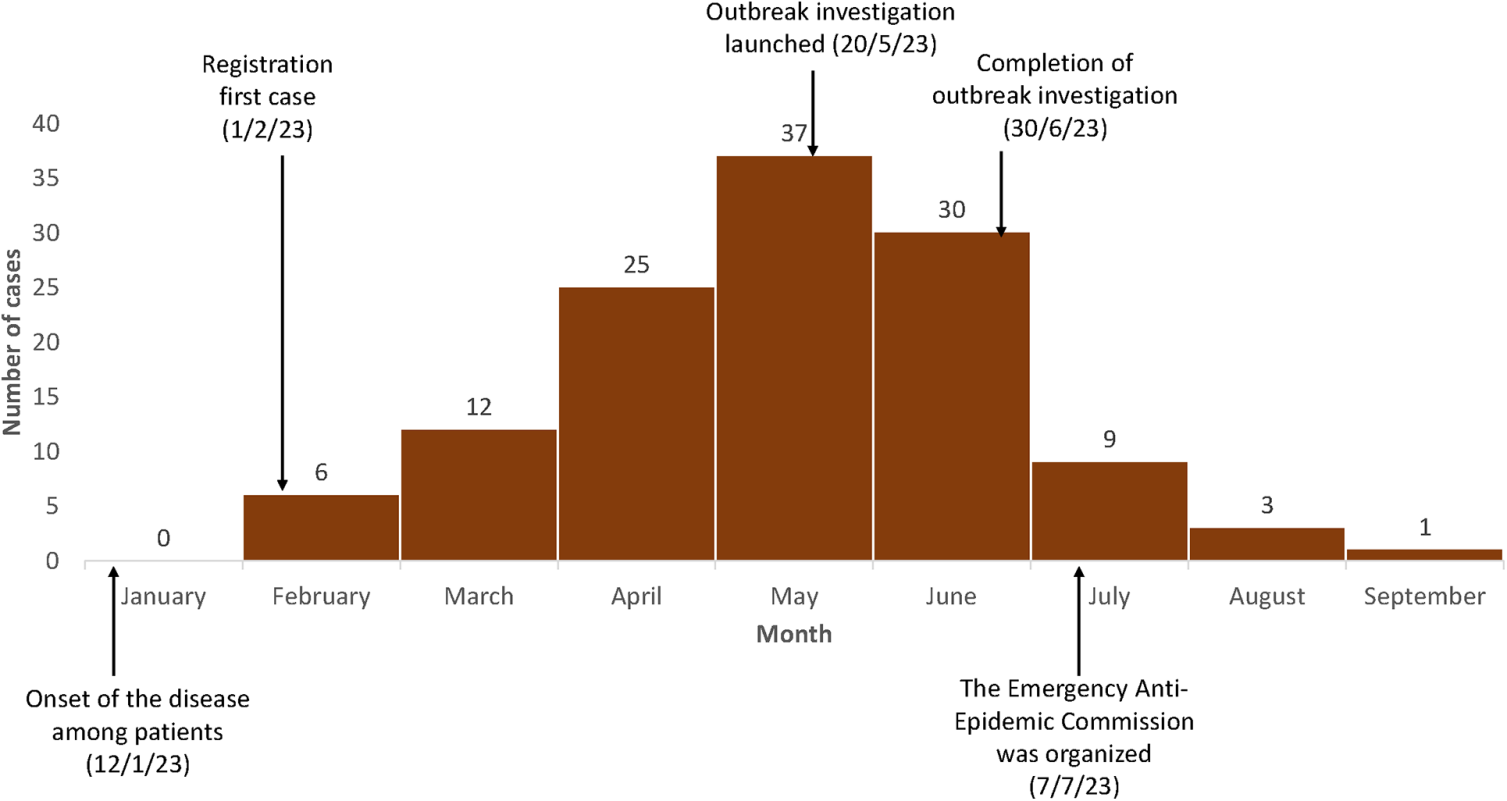
Epidemic curve of monthly cases of brucellosis in a remote village experiencing an outbreak, Tajikistan, January to September 2023 (*N* = 123). Only cases before June 15th are include in the case-control study.

The first case in the district was reported in early February. The outbreak peaked in May 2023 with 37 case-patients (Figure 2). By the end of the outbreak, 123 case-patients had been detected, of whom 57 (46%) were from the same village. An outbreak response was initiated in mid-June, and there was a sharp drop in cases following the investigation and response. One case-patient was reported in September.

Among the 57 cases from the same village included in our study, the average time from symptom onset to diagnosis was 19 days (range, 3–105 days). One case-patient (2%) was hospitalized, and the remaining case-patients received outpatient care. The most common symptoms were joint pain (95%), fever (84%), sudden weakness (collapse) (72%), night sweats (65%), headache (61%) and decreased appetite (40%). All patients recovered.

Among all participants, including both cases and controls (*n* = 171), 34% were male (Table 1). Median age was 34 years (interquartile range 23–47). Most (94%) had secondary education. Half (49%) were housewives and 18% were unemployed.

**Table 1:** Characteristics of cases and controls residing in a village experiencing a brucellosis outbreak, Tajikistan, 2023.

Characteristics	Total	Cases	Control	*p*-value^b^
	*N* = 171^a^	*N* = 57^a^	*N* = 114^a^	
Sex				0.05
Female	113 (66%)	32 (56%)	81 (71%)	
Male	58 (34%)	25 (44%)	33 (29%)	
Age				0.08
0–14	25 (15%)	7 (12%)	18 (16%)	
15–44	97 (57%)	39 (68%)	58 (51%)	
45+	49 (29%)	11 (19%)	38 (33%)	
Education				0.70
Above secondary education	6 (3.5%)	3 (5.3%)	3 (2.6%)	
Below school age	4 (2.3%)	1 (1.8%)	3 (2.6%)	
Secondary education	161 (94%)	53 (93%)	108 (95%)	
Occupation				0.14
Unemployed	30 (18%)	13 (23%)	17 (15%)	
Homemaker	84 (49%)	27 (47%)	57 (50%)	
Retired	9 (5.3%)	0 (0%)	9 (7.9%)	
Child	35 (20%)	12 (21%)	23 (20%)	
Others	13 (7.6%)	5 (8.7%)	8 (7.7%)	
Owned any livestock	164 (96%)	57 (100%)	107 (94%)	0.10
Owned sheep	125 (73%)	47 (82%)	78 (68%)	0.05
Owned goats	101 (59%)	42 (74%)	59 (52%)	<0.01^c^
Owned cattle	153 (89%)	53 (93%)	100 (88%)	0.30
Owned horses	5 (2.9%)	2 (3.5%)	3 (2.6%)	>0.90
Owned other livestock	77 (45%)	29 (51%)	48 (42%)	0.30
Had unvaccinated livestock	171 (100%)	57 (100%)	114 (100%)	
Had any recent livestock stillbirths	35 (20%)	15 (26%)	20 (18%)	0.20
Traveled outside the village <6 months	23 (13%)	9 (16%)	14 (12%)	0.75
Ate homemade kaymak	92 (54%)	51 (89%)	41 (36%)	<0.01^c^
Ate home-raised goat or sheep meat	149 (87%)	56 (98%)	93 (82%)	<0.01^c^
Drank local raw milk	116 (68%)	46 (81%)	70 (61%)	0.01^c^
Engaged in animal slaughter	36 (21%)	24 (42%)	12 (11%)	<0.01^c^
Engaged in animal care	29 (17%)	18 (32%)	11 (9.6%)	<0.01^c^

^a^ *n* (%).

^b^ Pearson's Chi-squared test; Fisher's exact test.

^c^ Significant difference, Chi-square *p*-value.

The majority of participants (96%) owned livestock. Specifically, 73% owned sheep, 59% owned goats, and 89% owned cattle. No livestock in the village had been vaccinated against brucellosis in the last 5 years. One-fifth (20%) of participants reported recent stillbirths among their livestock. The majority, 87%, had not traveled outside of their region in the last 6 months.

When asked about risk behaviors in the past 6 months, 54% reported consuming homemade kaymak (clotted cream from unpasteurized milk), 87% had consumed home-raised meat, and 68% drank village-produced milk. One-fifth (21%) had engaged in animal slaughter, 17%) in animal care.

### Comparison of cases and controls

Cases and controls did not differ significantly by sex, age, education, or occupation (Table 2). Ownership of livestock did not differ, but a greater proportion of cases than controls owned goats (74% vs. 52%, *p* < 0.01). Additionally, a greater proportion of cases than controls had eaten homemade kaymak (89% vs. 36%, *p* < 0.01), home-raised meat (98% vs. 82%, *p* < 0.01) or village-produced milk (81% vs. 61%, *p* < 0.01). Involvement in animal slaughter and animal care was also higher among cases than controls (42% vs. 11% and 32% vs. 10%, respectively, *p* < 0.01 for both).

**Table 2:** Factors associated with human brucellosis in a village experience an outbreak, Tajikistan, 2023.

Risk factors	Cases	Controls	OR	*p* ^b^	AOR	*p* ^b^
	*N* = 57	*N* = 114	(95% CI)		(95% CI)	
Consumed (ref. neither meat nor kaymak)	2 (4%)	41 (36%)	Ref		Ref	
Both meat^a^ and kaymak	38 (67%)	27 (24%)	28 (5.4–148)	<0.01	59 (4.3–798)	<0.01
Meat^a^ but not kaymak	13 (23%)	14 (12%)	17 (3.1–96)	<0.01	54 (4.0–731)	<0.01
Kaymak but not meat^a^	4 (7%)	32 (28%)	2.0 (0.3–12)	0.44	3.5 (0.2–50)	0.35
Engaged in animal slaughter (ref. not)	24 (42%)	12 (11%)	12 (3.6–40)	<0.01	36 (2.8–461)	<0.01
Engaged in animal care (ref. not)	18 (32%)	11 (10%)	23 (3.0–177)	<0.01	12 (1.0–147)	0.05
Consumed local raw milk (ref. not)	46 (81%)	70 (61%)	2.4 (1.2–4.9)	0.02	7.0 (1.5–34)	0.01

OR, crude odds ratios; CI, confidence interval; AOR, adjusted odds ratio, adjusted for all other risk factors in the table.

^a^ Home-raised goat or sheep.

^b^ Wald-test of logistic regression coefficients.

### Factors associated with brucellosis

Multivariable logistic regression showed that brucellosis was associated with consumption of both homemade kaymak and home-raised meat compared with consumption of neither (AOR = 59, 95% CI = 4.3–798, *p* < 0.01), with home-raised meat but no kaymak compared with neither (AOR = 54, 95% CI = 4.0–731, *p* < 0.01), and with involvement in animal slaughter compared with no involvement (AOR = 36, 95% CI = 2.8–461, *p* < 0.01) (Table 2).

## Discussion

In May 2023, a sharp increase in the incidence of brucellosis was observed in a small rural village in Tajikistan. The increase was associated with direct contact with unvaccinated livestock and consumption of their products. Effective measures were adopted in response to this outbreak, and cases subsided by September.

All participants in the study lived near livestock, none of which had been vaccinated against *Brucella* spp. in at least 5 years, and most participants owned livestock. Close contact with livestock is a major risk factor for brucellosis in humans (14–16). Most rural households in Tajikistan rely on the ownership of a small number of livestock for food consumption and for income. We found that more cases than controls in our study owned goats. In many countries, including in Tajikistan, goats and sheep are a particularly common source of human brucellosis (17, 18). Previous studies of goats in Tajikistan have found high seroprevalence of brucellosis, ranging from 5.5% to 6.7% (19).

None of the households in the village had vaccinated their livestock against brucellosis in the last 5 years. Vaccination of animals against brucellosis is critical to eliminating spread of disease in livestock (20, 21). Tajikistan ranks among the world's poorest countries, and nearly one-third of the population experiences food insecurity; rural Tajikistan is particularly affected by poverty (22). Rural communities not only lack the economic means to pay for vaccinating livestock, but also vaccines are not available in or near remote villages. The village in this outbreak was located in a mountainous area about 4 h from the nearest city.

Due to the favorable geographical conditions in the village, every year at the end of winter and beginning of spring, the surrounding areas of the village grow grass that is used as pastures for animals throughout the region. Residents of other neighboring villages lend their livestock to the residents of this village on a contractual basis until autumn. The residents of this village care for the cattle given on a contractual basis together with their own livestock. No passport or vaccination certificate is required when transferring animals. Village animals might have acquired *Brucella* during this period of increased contact with animals from other areas. Unfortunately, there were insufficient funds to test animals as part of this outbreak investigation.

The remoteness of villages, like the one from this outbreak, also makes its population heavily reliant on home- or locally- produced foods. We found that a high proportion of participants consumed home- or locally-grown food products, and that people who consumed homemade kaymak, home-grown goat or sheep meat, and locally-produced milk had increased odds of brucellosis. Importantly, people who ate both homemade kaymak and homemade meat had increased odds of brucellosis compared with those who ate neither, even after adjusting for consumption of raw milk, and engagement in animal slaughter and direct animal care. All of which are known risk factors for brucellosis. While meat consumption alone is not a risk factor, consumption of undercooked meat is a known risk factor for brucellosis (1). Kaymak is a homemade traditional cream, which is made with unpasteurized goat/sheep or cow milk. Kaymak is produced using a specialized equipment. The temperature is maintained between 30°C and 40°C throughout the preparation process, and the product is consumed raw. Consumption of unpasteurized dairy products from goats and/or cows are implicated in brucellosis outbreaks globally each year (14, 15, 23). Likewise, in our study, people who drank locally produced milk had increased odds of brucellosis, even when controlling for consumption of kaymak and meat. While we did not specifically ask whether the milk they had consumed was pasteurized or not, pasteurization is not common in rural areas of Tajikistan. In the village, due to reduced economic means, neighbors frequently share milk and other locally-produced dairy products with one another. Therefore, in such a setting, a single household with a sick animal could result in many households becoming sick.

Participants’ knowledge about methods of protection against brucellosis is very low. Since more than 90% of the participants had secondary education, after graduating from high school people were mainly engaged in agriculture and livestock farming. The lack of information about the symptoms of brucellosis and methods of its prevention was the reason that patients did not go to a medical facility on time. Previous studies have shown that insufficient knowledge about brucellosis further contributes to the spread of infection in the population and can lead to highly risky actions (24–26).

Our study is subject to some important limitations. Dairy and meat products were not tested using laboratory methods during the study due to budgetary constraints. Testing these products would have strengthened the findings of the study. Another limitation of the study was that due to a lack of laboratory capacity, blood samples were not tested for brucellosis species identification. Correct diagnosis and species identification are critical tools for targeted surveillance and control of brucellosis (27, 28). Our analysis is also limited by a lack of power to compare small sub-groups. Specifically, most cases consumed both kaymak and meat; and few consumed only kaymak. There is insufficient power to detect a statistical difference between those that only ate kaymak and those that had no kaymak and no meat. Our questionnaire did not include a question about the type of local milk participants had consumed, specifically, it was not asked if the milk was unpasteurized and if it was cow or goat milk. However, consumption of cow milk is more common, and home pasteurization of milk is uncommon. Lastly, the study is an outbreak investigation of a single village, the results are not generalizable beyond the village of study. Nevertheless, the risk factors for brucellosis identified in this study, have also been identified in other brucellosis outbreaks around the world. Brucellosis prevention measures can be applied to other regions to prevent brucellosis outbreaks.

Results of the study were shared with the head of the administration of the district and region, and brought to the attention of the national Ministry of Health and Social Protection of the Population of the Republic of Tajikistan. The Ministry of Health, established an Emergency Epidemic Control Commission in the district, which included all interested structures in order to prevent the spread of infection and carry out anti-epidemic measures, but the study team was not directly involved in this process.

Epidemic control measures taken by the emergency team included slaughter of diseased animals. The team also informed the public about the characteristics of brucellosis, proper animal care and safe consumption of dairy and meat products, including avoiding eating undercooked meat. The brucellosis outbreak was contained by August. To achieve long-term prevention, a brucellosis vaccination campaign for livestock was initiated in the village and district, and veterinary services increased monitoring of the quality of milk and dairy products. Over 5,000 animals were vaccinated in the district by the end of 2023.

## Conclusions

Our study showed that brucellosis remains an important public health problem in northwest Tajikistan. Contact with unvaccinated livestock and consumption of their products played a key role in this outbreak in a remote mountainous village. Cases were likely higher than reported because one in ten randomly selected controls tested positive for a previous infection. Following our investigation, a brucellosis prevention education campaign and animal vaccination campaigns were carried out in the village and surrounding district. No more brucellosis cases were reported after September 2023.

## Data Availability

The raw data supporting the conclusions of this article will be made available by the authors, without undue reservation.

## Footnotes

^1^See e.g., 45 C.F.R. part 46, 21 C.F.R. part 56; 42 U.S.C. §241(d); 5 U.S.C. §552a; 44 U.S.C. §3501 et seq.
